# Personalized prediction of live birth prior to the first in vitro fertilization treatment: a machine learning method

**DOI:** 10.1186/s12967-019-2062-5

**Published:** 2019-09-23

**Authors:** Jiahui Qiu, Pingping Li, Meng Dong, Xing Xin, Jichun Tan

**Affiliations:** 1 Reproductive Medical Center of Gynecology and Obstetrics Department, Shengjing Hospital of China Medical University, Shenyang, Liaoning China; 2 Key Laboratory of Reproductive Dysfunction Diseases and Fertility Remodeling of Liaoning Province, Shenyang, Liaoning China

**Keywords:** Prediction model, Cumulative live birth, IVF/ICSI, Machine learning

## Abstract

**Background:**

Infertility has become a global health issue with the number of couples seeking in vitro fertilization (IVF) worldwide continuing to rise. Some couples remain childless after several IVF cycles. Women undergoing IVF face greater risks and financial burden. A prediction model to predict the live birth chance prior to the first IVF treatment is needed in clinical practice for patients counselling and shaping expectations.

**Methods:**

Clinical data of 7188 women who underwent their first IVF treatment at the Reproductive Medical Center of Shengjing Hospital of China Medical University during 2014–2018 were retrospectively collected. Machine-learning based models were developed on 70% of the dataset using pre-treatment variables, and prediction performances were evaluated on the remaining 30% using receiver operating characteristic (ROC) analysis and calibration plot. Nested cross-validation was used to make an unbiased estimate of the generalization performance of the machine learning algorithms.

**Results:**

The XGBoost model achieved an area under the ROC curve of 0.73 on the validation dataset and showed the best calibration compared with other machine learning algorithms. Nested cross-validation resulted in an average accuracy score of 0.70 ± 0.003 for the XGBoost model.

**Conclusions:**

A prediction model based on XGBoost was developed using age, AMH, BMI, duration of infertility, previous live birth, previous miscarriage, previous abortion and type of infertility as predictors. This study might be a promising step to provide personalized estimates of the cumulative live birth chance of the first complete IVF cycle before treatment.

## Background

Infertility is a disease characterized by the failure to establish a clinical pregnancy after 12 months of regular, unprotected sexual intercourse [1]. Of reproductive-aged couples worldwide, 8–12% are struggling with it according to estimates [2]. Infertility is not only a health problem but also a psycho-social and public health issue. The demand for in vitro fertilization (IVF) is increasing, with over 8 million babies born through IVF or other assisted reproductive technology treatments since the world’s first in 1978 [3]. This does not guarantee success, with some couples remaining childless after several IVF cycles. Women undergoing IVF face greater risks of maternal and neonatal complications. e.g. ovarian hyperstimulation syndrome (OHSS), thrombus-embolism, infection, and abdominal bleeding [4]. Moderate-to-severe OHSS, which is potentially life-threatening, occurs in approximately 3–8% of cycles [5]. Furthermore, IVF itself is costly and few governments subsidize assisted reproductive technology (ART) cycles within their national health insurance schemes. In most countries, the cost of a single cycle is more than half of an average individual’s annual income. Many infertile couples incur catastrophic expenditure in order to pay for the high-cost of IVF treatment [6]. Consequently, an evidence-based tool of the probability of a successful live birth before an IVF treatment to assist with patient counseling is needed in clinical practice.

Numerous attempts have been made to estimate the live birth chance for women undergoing IVF treatment. However, the existing pre-treatment prediction models do not perform as well as expected and the value of the c-statistic used as a measure of discrimination is low. In addition, previous live birth prediction models have focused on fresh-embryo transfer instead of giving sufficient consideration to frozen-embryo transfer [7]. The newest and most accepted pre-treatment model is the McLernon model, which provides an individualized estimate cumulative chance of a live birth over a complete IVF cycle both before treatment and after the first fresh-embryo transfer [8, 9]. A complete IVF cycle refers to the fresh cycle and all the following freeze–thaw cycles from one round of ovarian stimulation. The model was built using data from the Human Fertilization and Embryology Authority database in the UK, which is the longest running database of its kind in the world and has recorded data since 1991. However, some crucial baseline factors which might be predictive factors for live birth are not included in the database. Due to this limitation, the McLernon model did not take factors such as body mass index (BMI) and anti-Müllerian hormone (AMH) into account. There has been extensive research regarding BMI and AMH for live birth prediction. A meta-analysis clearly demonstrates that female obesity negatively and significantly impacts live birth rates following IVF [10]. A systematic review confirmed that AMH is an indirect estimate of ovarian reserve has some value in predicting live birth and may be a predictor of live birth in women undergoing assisted conception [11]. Furthermore, in the past 40 years, ART has developed greatly from traditional IVF to intracytoplasmic sperm injection (ICSI) and preimplantation genetic screening (PGS)/preimplantation genetic diagnosis (PGD). There is no doubt that many future innovations will be made in the ART field. It is therefore important for all existing models to be updated regularly, which is a difficult and time-consuming process.

Machine learning is the science focusing on how computers learn from data without being explicitly programmed [12]. There is no universally accepted and clear definition of machine learning. Machine learning based algorithms are often categorized as supervised and unsupervised. Supervised machine learning is a process in which the model is trained with fully labeled and classified data. In contrast, unsupervised machine learning leaves the algorithms to discover on their own the underlying structure within unlabeled data. The machine learning based algorithms with strong data processing ability have become a promising methodology for clinical decision making and medicine study, including clinical prediction, radiology, surgery, drug discovery and pharmacokinetic prediction [13–15]. In the field of reproduction science, machine learning has been applied in areas including embryo scoring and prediction of implantation rate after blastocyte transfer [16, 17]. Compared with traditional statistical analysis methods relying on a predetermined equation as a model, machine learning can take full account of the interactions among variations and incorporate new data to update algorithms [18]. Hence, using a machine learning algorithm might improve the prediction performance of a pre-treatment model and make it easy to update. To date, no study has applied machine learning to live birth prediction before an IVF treatment.

The main objective of this study is to develop and assess a machine learning based clinical prediction model for estimating the cumulative live birth chance of the first complete IVF cycle using pre-treatment variables including BMI and AMH.

## Methods

### Data acquisition

Women undergoing IVF (including ICSI) at the Reproductive Medical Center of Shengjing Hospital, China from January 2014 to December 2018 were retrospectively reviewed. For a woman, the first fresh cycle and all following freeze–thaw cycles from the same ovarian stimulation were considered. Exclusion criteria included previous IVF/ICSI attempts, using frozen gametes, donor oocyte/sperm cycles and PGD/PGS cycles. Clinical data and patients’ baseline information were extracted from the patient database used in the fertility center. All data were de-identified and used with unique patient identifier codes. The primary outcome was ongoing pregnancy leading to at least one live birth according to the World Health Organization.

### Derivation and validation of models

Four supervised machine learning algorithms were respectively considered to build the predictive models: logistic regression, random forest, extreme gradient boosting (XGBoost) and support vector machine (SVM). All algorithms can deal with classification problems. Logistic regression is a common supervised classification algorithm with a nice probabilistic interpretation. The SVM is good at high dimension data, making it popular for many machine learning practitioners. Compared with logistic regression and SVM, XGBoost and random forest are both ensemble techniques that produce a prediction model by constructing a set of weaker learners, typically decision trees, and predict by combining the outcomes of each individual tree. The biggest difference lies in the way the trees are built. The random forest trains each tree independently by random sampling from the data. The XGBoost builds trees sequentially with each new tree trying to correct for the errors in the previous tree.

Getting pregnant is a complex process. Having a live birth after IVF treatment is influenced by a range of factors. In this study, the original dataset included more than 100 variables. Not all variables had a significant prediction effect on live birth and the candidate predictors should be clearly defined, standardized, and reproducible. Therefore, feature selection was performed based on subject knowledge, on pathophysiological mechanisms, or the results of previous studies and guidelines. We encoded categorical features using a one-hot encoding scheme. After data processing, 70% of the dataset was randomly selected as a training set for prediction model establishment, and the remaining 30% was used for validation. A stratified random sampling method was employed to ensure that the proportions of live birth and no live-birth cases were the same in both the training and validation sets as in the original dataset. Grid-search with k-fold cross-validation (k = 5) was used to find the optimal hyperparameters of the four classification classifiers mentioned above. The training set was subdivided into k folds. Each time, k − 1 folds were used for training and the remaining one was for validation. For each algorithm, models with different hyperparameters were scored by their mean accuracy. We chose the hyperparameter set that maximized the mean accuracy and fitted the model with the whole training dataset respectively. To evaluate the performance of each machine learning classifier, we assessed discrimination and calibration, which are widely used in prediction model validation. The receiver operating characteristic (ROC) curve and the calibration plot of the four chosen models for the validation set were adopted as a measure of discrimination and calibration.

In order to obtain an unbiased estimate of the generalization performance of these four classifiers to new patients, we also used repeated nested cross-validation to avoid sampling bias and data overfitting [19]. There are two cross-validation cycles in nested cross-validation. The outer K1 fold cross-validation where the dataset was split into the training validation set and the test set was to estimate the generalization performance of the learning pipeline. The inner K2 fold cross-validation was to tune hyperparameters and train models independently on the training validation set. In this study, we set K1 = K2 = 5 and repeated it 11 times.

### Statistical analysis and machine learning platform

Patients’ characteristics were described as counts and percentages or as means and standard deviations for categorical and continuous variables, respectively. Differences in the distribution of variables between live birth and no live-birth patients were assessed by Chi square test for categorical variables and by Student’s *t* test for continuous variables. All statistical analysis was conducted using SPSS version 22. Machine learning algorithms and plotting were performed with Python version 2.7. Python Sklearn and XGBoost packages were used.

## Results

### Basic characters

Electronic medical records from 9256 women were collected and reviewed in strict accordance with the set criteria. We excluded women with no pregnancy outcomes follow-up (n = 1732) and incomplete cases with missing data in any study feature (n = 336). Finally, a total of 7188 women’s first complete IVF/ICSI cycles were included in this study. There were 2797 (39%) women confirmed with live birth. Table 1 summarizes the baseline characters of the study population. Mean age at the time of IVF treatment was 32.66 years. The average duration of infertility was 4.2 years. Mean AMH and BMI were 4.05 ng/ml and 23.32 kg/m^2^, respectively. There were significant differences between the groups of patients who did and did not achieve a live birth.

**Table 1:** Descriptive statistics of the study population

Characteristics	Total N = 7188 Mean ± SD/N (%)	Live birth N = 2797 Mean ± SD/N (%)	No live birth N = 4391 Mean ± SD/(%)	P-value
Age (years)	32.66 ± 4.96	30.63 ± 3.59	33.96 ± 5.26	< 0.001
AMH (ng/ml)	4.05 ± 3.61	5.25 ± 4.03	3.29 ± 3.10	< 0.001
Duration of infertility	4.2 ± 3.19	3.80 ± 2.64	4.45 ± 3.47	< 0.001
BMI (kg/m^2^)	23.32 ± 3.76	23.14 ± 3.63	23.44 ± 3.83	0.001
Previous live birth	647 (9.0)	131 (4.7)	516 (11.9)	< 0.001
Previous miscarriage	1544 (21.5)	464 (16.6)	1080 (24.6)	< 0.001
Previous abortion	998 (13.9)	356 (12.7)	642 (14.6)	0.024
Type of infertility				
Tubal	3824 (53.2)	1546 (55.3)	2278 (51.9)	0.005
Anovulatory	1785 (24.8)	521 (18.6)	1264 (28.8)	< 0.001
Male factor	2960 (41.2)	1271 (45.4)	1689 (38.5)	< 0.001
Others	353 (4.9)	131 (4.7)	222 (5.1)	0.476
Unexplained	202 (2.8)	57 (2.0)	145 (3.3)	0.002

### Feature selection

The initial dataset included 108 variables (Additional file 1). Predictors were selected mainly according to previous studies and NICE clinical guidelines (The National Institute for Health and Care Excellence, UK). NICE clinical guidelines recommend female age, number of previous IVF treatment, previous pregnancy history, BMI and lifestyle factors can be used to predict IVF success. Moreover, the guidelines also demonstrate the important values of number of embryos transferred, ovarian reserve (AMH, AFC, FSH, etc.), duration of infertility, cause of infertility, number of oocytes retrieved and number of embryos available on prediction of IVF success [20]. Age and BMI are associated with lower pregnancy chances of IVF due to decreased both quality and quantity of oocytes. AMH is an indirect estimate of ovarian reserve. Studies show pregnancy rates were lower in couples with a longer duration of subfertility both in IVF and natural conception, even after adjustment for age [21]. Personal pregnancy history and type of infertility can indirectly reflect many complex factors of pregnancy including semen quality and endometrium receptivity, etc. As our model is for pretreatment counseling, we could only use the clinical data that obtained before starting an IVF procedure. Taken together, we selected age, AMH, BMI, duration of infertility, previous live birth, previous miscarriage, previous abortion and type of infertility as predictors. Type of infertility was classified into tubal, anovulatory, male factor, unexplained and others (e.g. endometriosis, fibroids).

### Model selection

**Fig. 1:**
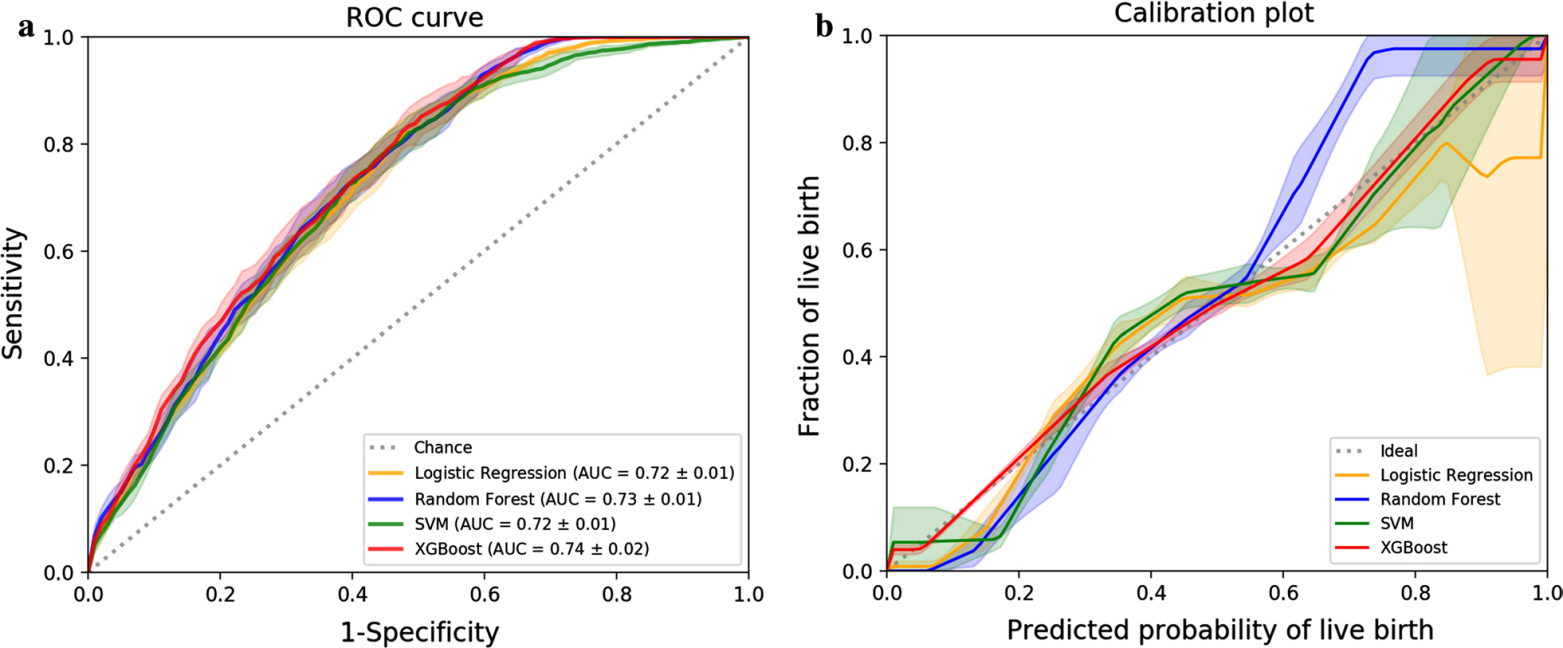
Cross-validated model performance of four machine learning algorithms on the training dataset. **a** Receiver operating characteristic curve plot. **b** Calibration plot. AUC indicates area under the curve. Shaded areas depict the standard deviation across different folds in a five-fold cross-validation

We used grid-search combined with five-fold cross-validation to optimize the best model parameters. Figure 1 illustrates the ROC and calibration analysis of four classifiers with optimal hyperparameters on the training dataset using five-fold cross-validation. The results for the area under the ROC curve (AUC) and calibration are shown by the average of these values over five different folds.

### Model performance

**Fig. 2:**
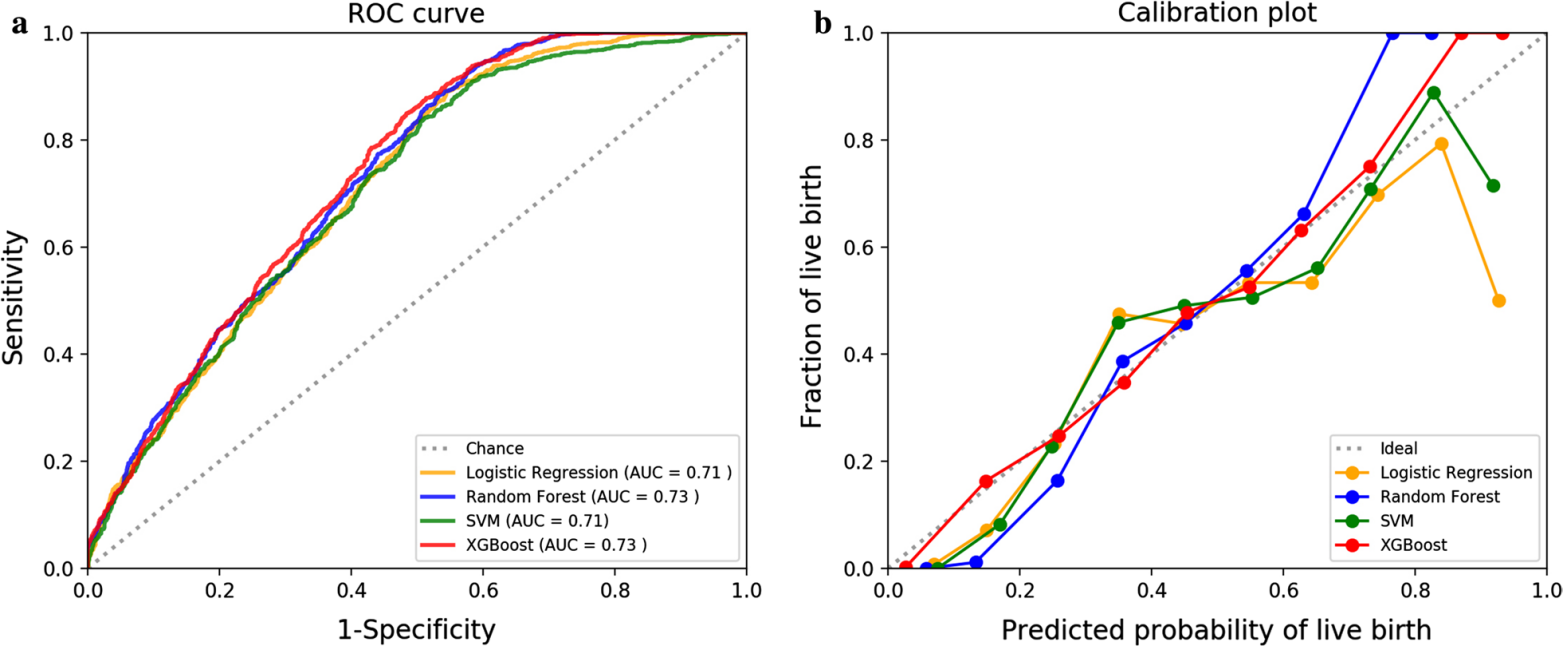
Final model performances of four machine learning algorithms on the validation dataset. **a** Receiver operating characteristic curve plot. **b** Calibration plot. AUC indicates area under the curve

The ROC curves of four selected models for the validation dataset are shown in Fig. 2a. The four models showed small differences, but XGBoost and random forest had slightly higher AUC of 0.73. Calibration of the four models in 10 bins is shown in Fig. 2b. The XGBoost exhibited the best calibration among all models, although it tended to underestimate the probability for high-probability patients. In conclusion, XGBoost provided the most accurate and robust prediction on the cumulative live birth chance for the first complete IVF cycles.

**Fig. 3:**
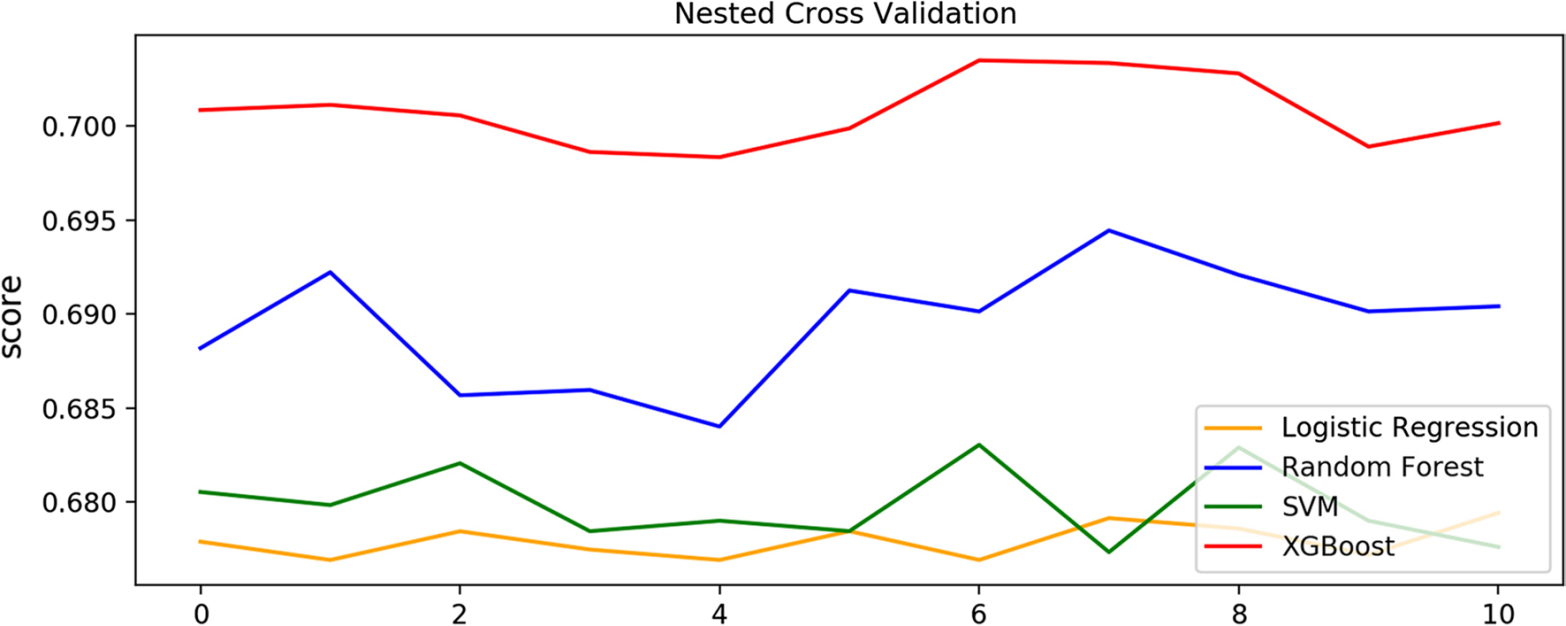
Nested cross-validation. Nested cross-validation outcomes for 11 times

Nested cross-validation was performed for the whole dataset. A nested cross-validation procedure always provides an unbiased estimate error which is very close to that obtained on the new data in practical application. Figure 3 shows the accuracy scores of each trial. Consistent with the aforementioned result, XGBoost provided the best performance, with an average accuracy score of 0.70, compared to 0.69, 0.68 and 0.68 for random forest, SVM and logistic regression, respectively.

## Discussion

**Fig. 4:**
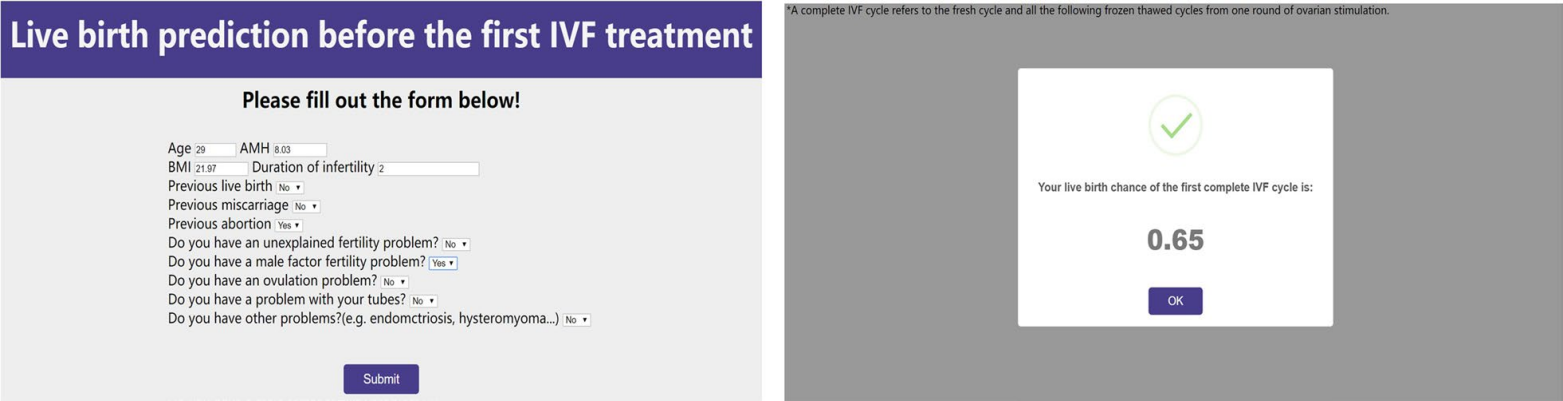
Live birth prediction tool (https://lbprediction.herokuapp.com)

In this study, we developed four machine learning models to predict the live birth probability for the first complete IVF attempt using pre-treatment patient variables. The XGBoost model achieved the best performance for discrimination and calibration. For example, using the XGBoost prediction model, an example is a 29-year-old woman with AMH of 8.03 ng/ml and BMI of 21.97 who had suffered from infertility caused by the male factor for 2 years. She had an abortion once before marriage. Now she has a 65% probability of achieving a live birth after the first complete cycle of IVF (Fig. 4). A web tool was developed for practice (https://lbprediction.herokuapp.com), which made it easily accessible for both patients and clinicians.

### Study strengths and limitations

Machine learning algorithms have been successfully used in many complex scenarios. Unique advantages of machine learning include flexibility and scalability, which make it suitable for many tasks, such as risk stratification, diagnosis and classification, and survival predictions [22]. XGBoost is a newly developed algorithm following the principle of gradient boosting, but with higher calculating speed and accuracy [23]. Many clinical prediction models have been established using XGBoost, in which XGBoost performed significantly better than traditional statistical approaches [24–26]. To our knowledge, that is the first published pre-treatment live birth prediction model based on a machine learning algorithm for women preparing to accept an IVF treatment. It represents a good attempt at combining a machine learning algorithm with reproduction big data. In this study, the XGBoost model achieved AUC = 0.73 for the validation dataset. Although this was not an ideal score, to date, no existing model has achieved a higher score. In addition, our model accounted for BMI and AMH. As the univariate analysis result showed, BMI and AMH were significantly associated with live birth. A recent external validation study of McLernon models suggested that the addition of AMH and body weight to McLernon models could improve the c-statistic [27]. Furthermore, on the basis of a self-updating ability of machine learning, we can easily update the XGBoost model by continuously providing it with the newest data.

Several limitations of this study deserve to be mentioned. First, the study was performed based on data derived from a single center. It is possible that the model might be suitable for the stratified population, which may limit the generalization of the model to other populations. Fortunately, our study showed the ability of XGBoost for predicting live birth. We only need to fit the model with multicenter data or build more center-specific models using single center data. Second, our model can only be used for couples who have never accepted IVF treatment, which limits the application of the model. Third, we failed to account for family genetic history and lifestyle factors such as smoking status, alcohol and caffeine consumption due to the limitation of dataset.

### Clinical implications

In clinical practice, when clinicians provide counseling on live birth chances for an IVF treatment, they rely on the mean success rate of the fertility center or their experience mainly by age, which is inaccurate. This study provides an online calculator that is adjusted for every patient’s easily measurable predictors to personalized estimates of the cumulative live birth chance of the first complete IVF cycle before treatment. The calculator will assist with patient counselling and help couples accepting IVF treatment prepare emotionally, thus avoiding deep disappointment caused by high expectations. Currently, the tool is not advised to be used on decision making around whether or not should couples accept an IVF treatment. The model was established based on very limited predictors obtained before an IVF treatment. Pregnancy is a dynamic and ongoing process. Given the complexity of a full term delivery we appreciate successful live birth depends on more than the factors in this model alone. There are many other confounders that have an impact at different time points. For example, factors such as number of oocytes retrieved, quality and number of embryos transferred, endometrial thickness on embryo transferred day which are crucial factors during an IVF treatment, were not taken into account in this study. Therefore, the model might give a false hope to couples who have a strong desire for a baby. It’s important for clinicians and patients to be aware of the limitations when using it.

## Conclusions

In conclusion, we established a pre-treatment live birth prediction model for the first complete IVF cycle based on a machine learning algorithm, XGBoost. To further improve the performance of our XGBoost model, in subsequent research, we will continuously collect clinical data for real-time updating of the model. More functions will be added to the model, such as prediction of birth defects, preterm birth, low birth weight and other adverse pregnancy outcomes. A randomized controlled trial will be performed to evaluate whether the model improves satisfaction of couples accepting IVF treatment.

## Supplementary information

**Additional file 1.** Variables in the initial dataset.

## Data Availability

The datasets used and/or analyzed during the current study are available from the corresponding author on reasonable request.

## Abbreviations

IVF: in vitro fertilization
ART: assisted reproductive technology
ROC: receiver operating characteristic
AUC: receiver operating characteristic curve

## References

[1] Zegers-Hochschild F, Adamson GD, Dyer S, Racowsky C, de Mouzon J, Sokol R (2017). The international glossary on infertility and fertility care, 2017. Hum Reprod.

[2] Vander Borght M, Wyns C (2018). Fertility and infertility: definition and epidemiology. Clin Biochem.

[3] Crawford GE, Ledger WL (2019). In vitro fertilisation/intracytoplasmic sperm injection beyond 2020. BJOG.

[4] Braat DD, Schutte JM, Bernardus RE, Mooij TM, van Leeuwen FE (2010). Maternal death related to IVF in the Netherlands 1984–2008. Hum Reprod.

[5] Mourad S, Brown J, Farquhar C (2017). Interventions for the prevention of OHSS in ART cycles: an overview of Cochrane reviews. Cochrane Database Syst Rev.

[6] Inhorn MC, Patrizio P (2015). Infertility around the globe: new thinking on gender, reproductive technologies and global movements in the 21st century. Hum Reprod Update.

[7] Dhillon RK, McLernon DJ, Smith PP, Fishel S, Dowell K, Deeks JJ (2016). Predicting the chance of live birth for women undergoing IVF: a novel pretreatment counselling tool. Hum Reprod.

[8] McLernon DJ, Steyerberg EW, Te Velde ER, Lee AJ, Bhattacharya S (2016). Predicting the chances of a live birth after one or more complete cycles of in vitro fertilisation: population based study of linked cycle data from 113,873 women. BMJ.

[9] McLernon DJ, Steyerberg EW, Te Velde ER, Lee AJ, Bhattacharya S (2018). An improvement in the method used to assess discriminatory ability when predicting the chances of a live birth after one or more complete cycles of in vitro fertilisation. BMJ.

[10] Sermondade N, Huberlant S, Bourhis-Lefebvre V, Arbo E, Gallot V, Colombani M (2019). Female obesity is negatively associated with live birth rate following IVF: a systematic review and meta-analysis. Hum Reprod Update.

[11] Iliodromiti S, Kelsey TW, Wu O, Anderson RA, Nelson SM (2014). The predictive accuracy of anti-Mullerian hormone for live birth after assisted conception: a systematic review and meta-analysis of the literature. Hum Reprod Update.

[12] Deo RC (2015). Machine learning in medicine. Circulation.

[13] Handelman GS, Kok HK, Chandra RV, Razavi AH, Lee MJ, Asadi H (2018). eDoctor: machine learning and the future of medicine. J Intern Med.

[14] Rahimian F, Salimi-Khorshidi G, Payberah AH, Tran J, Ayala Solares R, Raimondi F (2018). Predicting the risk of emergency admission with machine learning: development and validation using linked electronic health records. PLoS Med.

[15] Ross EG, Jung K, Dudley JT, Li L, Leeper NJ, Shah NH (2019). Predicting future cardiovascular events in patients with peripheral artery disease using electronic health record data. Circ Cardiovasc Qual Outcomes.

[16] Santos Filho E, Noble JA, Poli M, Griffiths T, Emerson G, Wells D (2012). A method for semi-automatic grading of human blastocyst microscope images. Hum Reprod.

[17] Blank C, Wildeboer RR, DeCroo I, Tilleman K, Weyers B, de Sutter P (2019). Prediction of implantation after blastocyst transfer in in vitro fertilization: a machine-learning perspective. Fertil Steril.

[18] Waljee AK, Higgins PD (2010). Machine learning in medicine: a primer for physicians. Am J Gastroenterol.

[19] Koutsouleris N, Kahn RS, Chekroud AM, Leucht S, Falkai P, Wobrock T (2016). Multisite prediction of 4-week and 52-week treatment outcomes in patients with first-episode psychosis: a machine learning approach. Lancet Psychiatry.

[20] National Collaborating Centre for Women’s and Children’s Health (UK). Fertility: assessment and treatment for people with fertility problems. London: Royal College of Obstetricians & Gynaecologists; 2013 Feb. (NICE Clinical Guidelines, No. 156.) https://www.ncbi.nlm.nih.gov/books/NBK247932/. 25340218

[21] van Loendersloot L, Repping S, Bossuyt PM, van der Veen F, van Wely M (2014). Prediction models in in vitro fertilization; where are we? A mini review. J Adv Res.

[22] Ngiam KY, Khor IW (2019). Big data and machine learning algorithms for health-care delivery. Lancet Oncol.

[23] Chen T, Guestrin C, editors. Xgboost: A scalable tree boosting system. In: Proceedings of the 22nd acm sigkdd international conference on knowledge discovery and data mining; 2016: ACM.

[24] Khemasuwan D, Sorensen J, Griffin DC (2018). Predictive variables for failure in administration of intrapleural tissue plasminogen activator/deoxyribonuclease in patients with complicated parapneumonic effusions/empyema. Chest.

[25] Xiao J, Ding R, Xu X, Guan H, Feng X, Sun T (2019). Comparison and development of machine learning tools in the prediction of chronic kidney disease progression. J Transl Med.

[26] Zhang Z, Ho KM, Hong Y (2019). Machine learning for the prediction of volume responsiveness in patients with oliguric acute kidney injury in critical care. Crit Care.

[27] Leijdekkers JA, Eijkemans MJC, van Tilborg TC, Oudshoorn SC, McLernon DJ, Bhattacharya S (2018). Predicting the cumulative chance of live birth over multiple complete cycles of in vitro fertilization: an external validation study. Hum Reprod.

