# Supplementary material for: Personalized prediction of live birth prior to the first in vitro fertilization treatment: a machine learning method
Source: J Transl Med. 2019 Sep 23;17:317. doi: 10.1186/s12967-019-2062-5 (PMC6757430; doi:10.1186/s12967-019-2062-5)
Supplement: Supplementary file 1 — Additional file 1. Variables in the initial dataset. [file 12967_2019_2062_MOESM1_ESM.docx]

ID

Female age

Male age

Female ethnicity

Male ethnicity

Female BMI

Male BMI

Female education

Male education

AMH

duration of infertility

previous live birth

previous miscarriage

previous abortion

type of infertility

chromosome

hepatitis

tuberculosis

appendicitis

pelvic inflammation

operation history

cardiovascular disease

urinary infection

STD

menarche

menstrual blood volume

period

HGB

RBC

WBC

PLT

blood type

ESR

RH

PT

APTT

T3

T4

TSH

FT3

FT4

FSH

E2

P

PRL

LH

T

AFC

1st semen analysis

Abstinence time(d)

Semen volume(ml)

Sperm concentration(106/ml)

Progressive motility (PR, %)

Total motility (PR + NP, %)

Morphology (normal, %)

2nd semen analysis

Abstinence time(d)

Semen volume(ml)

Sperm concentration(106/ml)

Progressive motility (PR, %)

Total motility (PR + NP, %)

Morphology (normal, %)

3h semen analysis

Abstinence time(d)

Semen volume(ml)

Sperm concentration(106/ml)

Progressive motility (PR, %)

Total motility (PR + NP, %)

Morphology (normal, %)

TORCH

HBSAG

HBSAB

HBEAG

HBEAB

HBCAB

HBCAB-IGM

HCVAB

HIVAB

TP

GPT

GOT

SCR

BUN

chlamydia

mycoplasma

trichomoniasis

mold

NGH

temperature

P

R

BP

height

weight

nutrition

development

mental state

hair

skin

lymph node

breast

heart

lung

liver

spleen

kidney

trunk

vulva outlook

vagina morphology

Cervix

uterus morphology

ovary morphology
